# Thoracoabdominal Aortic Aneurysm in a HIV-positive Patient

**DOI:** 10.21470/1678-9741-2016-0045

**Published:** 2017

**Authors:** Márcio Luís Lucas, Ívia Binotto, Paulo Behar, Nilon Erling Jr., Eduardo Lichtenfels, Newton Aerts

**Affiliations:** 1 Department of Vascular Surgery at Santa Casa de Porto Alegre, RS, Brazil.; 2 Department of Infectious Diseases at Universidade Federal de Ciências da Saúde de Porto Alegre (UFCSPA), RS, Brazil.; 3 Department of Surgery at Universidade Federal de Ciências da Saúde de Porto Alegre (UFCSPA), RS, Brazil.

**Keywords:** Aortic Aneurysm/Surgery, HIV Infections, Aortic Diseases/Surgery, Cardiac Surgical Procedures

## Abstract

Advent of antiretroviral therapy has increased survival of patients with human immunodeficiency virus (HIV) infections, with the result that some of these patients now develop degenerative diseases, such as atherosclerotic aneurysms. Degenerative thoracoabdominal aortic aneurysm is rare in HIV patients. In this report, a 63-year-old male patient with HIV submitted to open repair of thoracoabdominal aortic aneurysm. The patient did not suffer any type of complication in the perioperative period and remained well in a 28-month follow-up period. In summary, open repair still remains a good alternative for aortic complex aneurysms even in HIV patients.

**Table:** 

Abbreviations, acronyms & symbols	
HIV TAAA	= Human immunodeficiency virus = Thoracoabdominal aortic aneurysm

## INTRODUCTION

Over recent decades, the advent of antiretroviral therapy has increased survival of patients with human immunodeficiency virus (HIV) infections, with the result that some of these patients now develop degenerative diseases, such as atherosclerotic aneurysms^[1]^. The technical advances achieved over recent decades have significantly improved the results of surgical treatment for thoracoabdominal aortic aneurysms (TAAA), which now achieves acceptable morbidity and mortality rates^[2,3]^. As a less invasive method, endovascular treatment has shown promising results for treatment for these complex aneurysms, although the long-term results are not yet well known and the reintervention rate is considerable^[4]^. Atherosclerotic TAAA in HIV-positive patients is rare and, for this reason, this article will describe the case of a patient with HIV infection and a symptomatic TAAA treated surgically.

## CASE REPORT

A male, 63-year-old patient was brought to the emergency service complaining of intense pain in the dorsal lumbar region, with onset approximately 12 hours previously and accompanied by nausea and vomiting. He was an HIV virus carrier and had been on treatment for 20 years, and was taking tenofovir, lamivudine and efavirenz at the time of presentation. He had a history of hypertension, under control, and had been a smoker since 13 years of age (40 cigarettes/day). He was an ex-user of illicit drugs (cocaine, marijuana and injectable drugs) and was hepatitis B and C positive. He was in good general health, free from fever, hemodynamically stable, and cardiac and pulmonary auscultation were normal. His abdomen was depressible and flaccid and he reported tenderness in response to deep palpation in the upper left quadrant and an expansive, pulsating mass was evident in the epigastrium, however, there were no signs of peritoneal irritation. Extremities were warm and perfused and all peripheral pulses were symmetrical and normal.

**Fig. 1:**
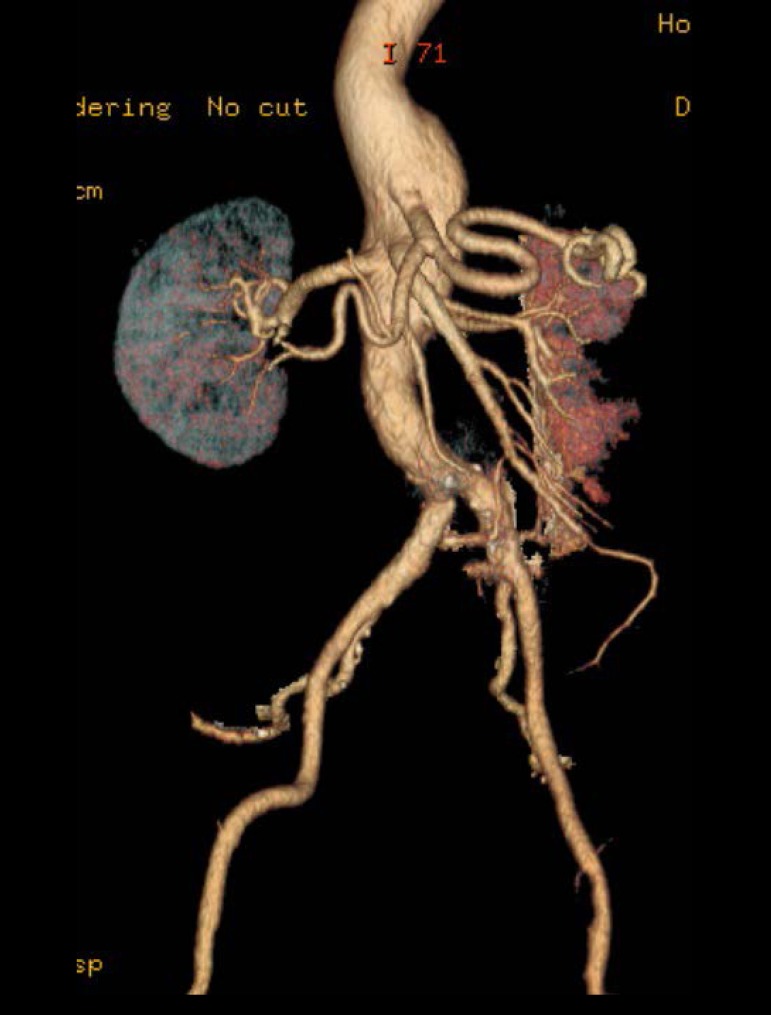
Preoperative reconstruction of a thoracoabdominal aortic aneurysm (TAAA) by CT angiography performed in a HIV-positive patient.

**Fig. 2:**
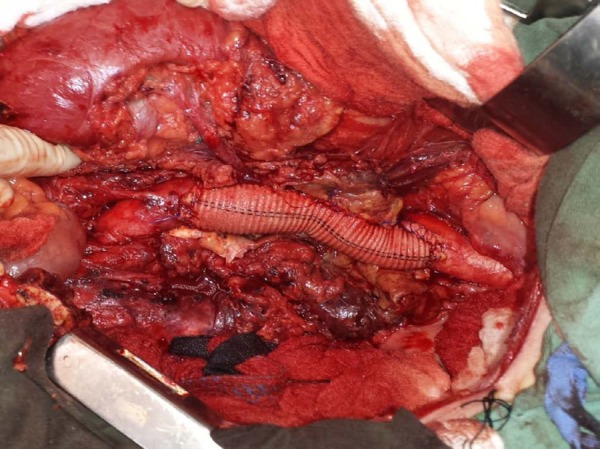
Operative view of aortic reconstruction during open repair of TAAA in a HIV-positive patient.

Laboratory test results were as follows: hemoglobin, 15.2g/dL; white blood cell count, 8950/mm^3^ (without left shift), erythrocyte sedimentation rate, 7 mm; creatinine, 0.9 mg/dL; CD4 T lymphocytes, 760 cells/mm^3^; and HIV viral load undetectable by polymerase chain reaction. Blood cultures were performed (3 samples) and later returned negative. Full abdominal echography showed an abdominal aortic aneurysm involving the renal arteries with a maximum diameter of approximately 5 cm. Thoracoabdominal angiotomography showed an aneurysmal dilatation of the distal thoracic aorta extending to the infra-renal abdominal aorta with maximum thoracic diameter of 6.2 cm and maximum abdominal diameter of 5.6 cm - type V TAAA according to the Crawford-Safi classification^[5]^ (Figure 1). Since symptoms were persistent, a preoperative clinical assessment was conducted and surgical treatment was prescribed. Access was achieved via thoraco-phreno-laparotomy, with full exposure of the aorta from the 6^th^ intercostal space to the aortoiliac bifurcation. An atherosclerotic aneurysm was identified, with atheromatous plaques, but free from any sign of inflammation or localized purulent secretion. The repair was accomplished by endoaneurysmorrhaphy with interposition of a 20 mm straight Dacron graft, using the clamp and go technique, with no bypasses or shunts. All visceral branches, including the left renal artery, were encompassed with a single suture line (Figure 2). The clamping time for proximal anastomosis was 9 minutes, mesenteric and renal ischemia duration was 20 minutes (island anastomosis of the graft at the orifices of the visceral vessels), and the time taken for distal anastomosis at the aortoiliac bifurcation was 11 minutes. Cell salvage was used to replace 500 mL of blood, from total bleeding of 800 mL, and blood transfusion was not needed.

The patient responded extremely well during the postoperative period, achieving hemodynamic stability without vasoactive drugs, was extubated approximately 6 hours after the end of surgery, had good diuresis (600 mL/12 hours), and was discharged from the intensive care unit within 36 hours. The patient did not suffer any type of complication in the ward and exhibited good conditions for hospital discharge on the 8^th^ postoperative day.

**Fig. 3:**
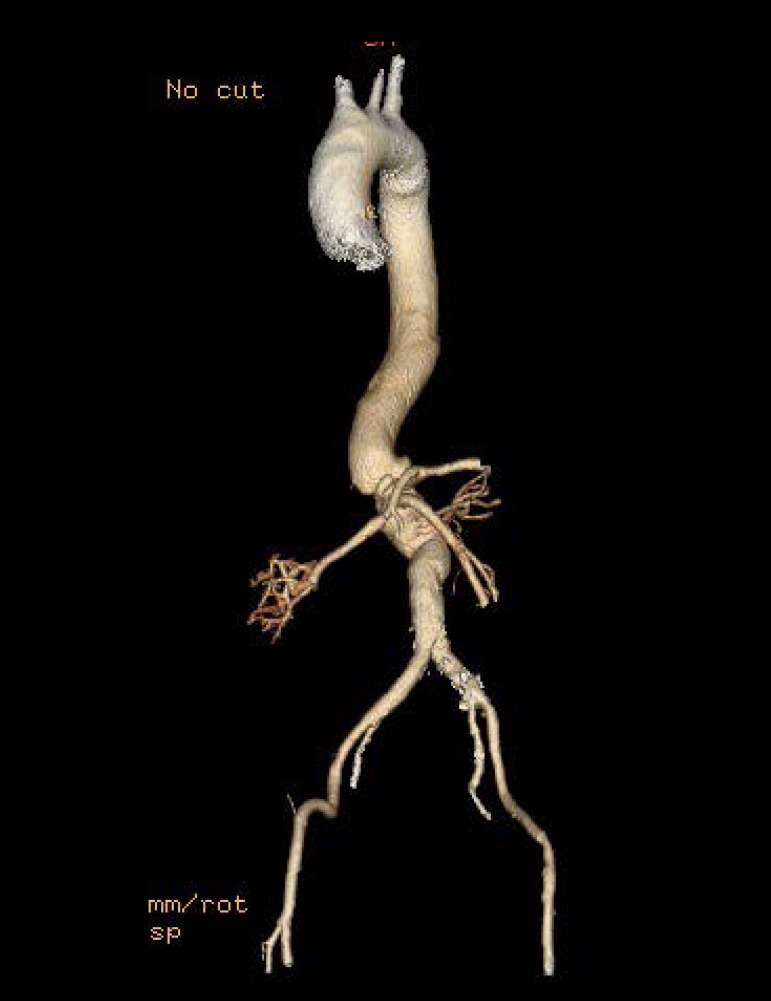
Postoperative CT angiography demonstrating a good result after open repair of TAAA in a HIV-positive patient.

Around 30 days after surgery, a control angiotomography showed patent visceral vessels and other features of the reconstruction were free from problems (Figure 3). The patient remained well during the 28-month follow-up period.

## DISCUSSION

Surgical treatment of TAAA is still a therapeutic challenge, although at some centers of excellence mortality rates are now acceptable, approaching 5%^[2]^. The risks involved in the procedure are greater in urgent situations, such as with symptomatic patients, and mortality rates can approach 35%^[6]^. Currently, branched/fenestrated endoprostheses appear promising for treatment of TAAA, although the long-term complications and mortality rates are not yet negligible^[4]^. Another treatment option is hybrid intervention, combining endovascular and open approaches to treat these aneurysms, however, the risk of perioperative death can also be high^[7]^.

The immunological status of patients infected with HIV has an influence on decision making. Therefore, in patients who have advanced HIV disease (*e.g.*, Acquired Immunodeficiency Syndrome), malnutrition and CD4 count below 200 cells/mm^3^, conservative treatment should be considered. In patients with CD4 levels higher than 500 cells/mm^3^, management should be equivalent to that for the population in general. For patients with intermediate CD4 values (from 200 to 500 cells/mm^3^), less-invasive treatment should be considered (e*.g.,* endovascular or extra-anatomic bypass)^[8]^. Our patient had an undetectable viral load and good immunological status, with a CD4 level of 760 cells/mm^3^, which is why we decided to offer open surgical treatment.

Development of aneurysms in HIV-positive patients may occur in a variety of different manners: as a result of direct action of the virus on the aorta wall, triggering an inflammatory process; bacterial infections of aorta with prior degeneration, characterizing a mycotic aneurysm; or degeneration of the aorta wall, resulting in atherosclerotic aneurysms, the appearance of which may be anticipated by changes to lipid metabolism caused by antiretroviral therapy^[9,10]^. Aneurysmal disease provoked by HIV is a distinct clinical entity, with no well-defined etiology, and which generally affects younger patients without risk factors for atherosclerotic disease^[9]^. It is generally associated with a reduction in CD4 levels and it can lead to adventitial damage and injury to the *vasa vasorum* by HIV^[9,10]^. These aneurysms are saccular and are found in atypical sites, tending to be multiple, with carotid and femoral arteries the most frequently involved^[8,10]^. Surgical treatment of patients with aneurysmal disease related to HIV has achieved limited results. A study by Ronns & Paruk^[11]^ described 226 HIV-positive patients with peripheral arterial disease and a mean age of 36 years, 111 treated for aneurysms and 115 for occlusive disease. The majority of these patients were treated with open surgery and the perioperative mortality rate was 9%^[11]^. In some cases, aneurysms may be associated with infectious processes, of which salmonella, tuberculosis and *Haemophilus influenzae* are the most frequent causes^[9,10]^.

However, HIV-positive patients may also have long survival and develop atherosclerotic aneurysms due to exposure to cardiovascular risk factors^[1]^. In the case described here, the history of smoking and hypertension, the patient’s age and his good immunological status led to designation of an atherosclerotic cause of the thoracoabdominal aneurysm. Mirza et al.^[12]^ also described a patient carrying HIV who was treated for an atherosclerotic aneurysm of the ascending aorta.

The duration of aortic clamping is linked with the risk of developing visceral complications, such as mesenteric ischemia and renal failure requiring hemodialysis, and also to increased mortality^[2]^. Visceral ischemia not exceeding 40 minutes can greatly reduce these risks. In our case, proximal, visceral and distal anastomoses were performed in 9, 11 and 11 minutes, respectively. In this context, constant training and improvement of the entire team is important, because results are also dependent on the number of procedures conducted.

## CONCLUSION

Antiretroviral therapy has significantly increased survival of HIV patients. As a result, cardiovascular diseases have become an important cause of later deaths among these patients. This is why knowledge of the evolution and results of treatment for different types of cardiovascular disease is essential when managing these patients. Certain ethical considerations are also involved in treatment of HIV-positive patients, and a judicious and considered decision should be made, reserving conservative treatment of patients with advanced HIV-related disease, while seropositive patients with favorable clinical conditions should be offered treatment similar to that offered to a seronegative patient.

**Table:** 

Authors’ roles & responsibilities	
MLL	Conception and study design; realization of operations; manuscript redaction or critical review of its content; final manuscript approval
IB	Conception and study design; realization of operations; manuscript redaction or critical review of its content; final manuscript approval
PB	Conception and study design; manuscript redaction or critical review of its content; final manuscript approval
NEJ	Conception and study design; realization of operations; manuscript redaction or critical review of its content; final manuscript approval
EL	Conception and study design; realization of operations; manuscript redaction or critical review of its content; final manuscript approval
NA	Conception and study design; realization of operations; manuscript redaction or critical review of its content; final manuscript approval

## References

[1] Gilbert JM, Fitch KV, Grinspoon SK (2015). HIV-related cardiovascular disease, statins, and the REPRIEVE
Trial. Top Antivir Med.

[2] Coselli JS, LeMaire SA, Preventza O, Cruz KI, Cooley DA, Price MD (2016). Outcomes of 3309 thoracoabdominal aortic aneurysm
repairs. J Thorac Cardiovasc Surg.

[3] Souza JM, Berlinck MF, Rojas SO, Senra DF, Oliveira PAF, Martins JRM (1991). Surgical treatment of thoracoabdominal aortic
aneurysms. Braz J Cardiovasc Surg.

[4] Verzini F, Loschi D, De Rango P, Ferrer C, Simonte G, Coscarella C (2014). Current results of total endovascular repair of thoracoabdominal
aortic aneurysms. J Cardiovasc Surg (Torino).

[5] Seidel AC, Miranda F, Marcantonio JM (2006). Ruptured thoracoabdominal aortic aneurysm in the right pleural
space. Braz J Cardiovasc Surg.

[6] Chiesa R, Civilini E, Melissano G, Logaldo D, Calliari FM, Bertoglio L (2009). Management of thoracoabdominal aortic aneurysms. HSR Proc Intensive Care Cardiovasc Anesth.

[7] Bakoyiannis C, Kalles V, Economopoulos K, Georgopoulos S, Tsigris C, Papalambros E (2009). Hybrid procedures in the treatment of thoracoabdominal aortic
aneurysms a systematic review. J Endovasc Ther.

[8] Botes K, van Marle J (2007). Surgical intervention for HIV related vascular
disease. Eur J Vasc Endovasc Surg.

[9] Nair R, Abdool-Carrim A, Chetty R, Robbs J (1999). Arterial aneurysms in patients infected with human
immunodeficiency virus a distinct clinicopathology entity?. J Vasc Surg.

[10] Bellows PH, Anaya-Ayala JE, Younes HK, Charlton-Ouw KM, Bismuth J, Davies MG (2010). Spontaneous regression of an abdominal aortic aneurysm in an
immunocompromised patient. Vasc Med.

[11] Robbs JV, Paruk N (2010). Management of HIV vasculopathy a South African
experience. Eur J Vasc Endovasc Surg.

[12] Mirza H, Patel P, Suresh K, Krukenkamp I, Lawson WE (2004). HIV disease and an atherosclerotic ascending aortic
aneurysm. Rev Cardiovasc Med.

